# *Ano5* modulates calcium signaling during bone homeostasis in gnathodiaphyseal dysplasia

**DOI:** 10.1038/s41525-022-00312-1

**Published:** 2022-08-18

**Authors:** Xin Li, Lei Wang, Hongwei Wang, An Qin, Xingjun Qin

**Affiliations:** 1 Department of Oral and Maxillofacial Head and Neck Oncology, Shanghai Ninth People’s Hospital, Shanghai Jiao Tong University School of Medicine, National Center for Stomatology, National Clinical Research Center for Oral Diseases, Shanghai Key Laboratory of Stomatology and Shanghai Research Institute of Stomatology, 200011 Shanghai, China; 2 Department of Oral and Maxillofacial Surgery, Affiliated Stomatology Hospital of Guangzhou Medical University, Guangzhou Key Laboratory of Basic and Applied Research of Oral Regenerative Medicine, 510182 Guangzhou, Guangdong China; 3 Department of Oral and Craniomaxillofacial Surgery, Shanghai Ninth People’s Hospital, Shanghai Jiao Tong University School of Medicine, 200011 Shanghai, China; 4 Shanghai Key Laboratory of Orthopedic Implants, Department of Orthopedics, Shanghai Ninth People’s Hospital, Shanghai Jiao Tong University School of Medicine, 200011 Shanghai, China

**Keywords:** Gene expression, Osteogenesis imperfecta, Gene regulation, Next-generation sequencing, Osteoporosis

## Abstract

*ANO5* encodes transmembrane protein 16E (TMEM16E), an intracellular calcium-activated chloride channel in the endoplasmic reticulum. Mutations in *ANO5* are associated with gnathodiaphyseal dysplasia (GDD), a skeletal disorder causing the jaw deformity and long bone fractures. However, the coordinated mechanism by which *ANO5* mediates bone homeostasis in GDD remains poorly defined. Here, we show that ablation of *Ano5* reduced intracellular calcium transients, leading to defects in osteogenesis and osteoclastogenesis and thus bone dysplasia. We found a causative de novo *ANO5* frameshift insertion mutation (p.L370_A371insDYWRLNSTCL) in a GDD family with osteopenia, accompanied by a decrease in TMEM16E expression and impaired RANKL-induced intracellular calcium ([Ca^2+^]_i_) oscillations in osteoclasts. Moreover, using *Ano5* knockout (KO) mice, we found that they exhibited low bone volume, abnormal calcium deposits, and defective osteoblast and osteoclast differentiation. We also showed that *Ano5* deletion in mice significantly diminished [Ca^2+^]_i_ oscillations in both osteoblasts and osteoclasts, which resulted in reduced WNT/β-Catenin and RANKL-NFATc1 signaling, respectively. Osteoanabolic treatment of parathyroid hormone was effective in enhancing bone strength in *Ano5* KO mice. Consequently, these data demonstrate that *Ano5* positively modulates bone homeostasis via calcium signaling in GDD.

## Introduction

Skeletal bone is continuously remodeled by the bone formation and resorption achieved by the coordinated action of osteoblasts and osteoclasts in both time and space^1^; thus, imbalances in bone homeostasis cause various skeletal disorders^2^. As we previously reported in an affected family, gnathodiaphyseal dysplasia (GDD) is a rare autosomal dominant (AD) skeletal syndrome involving skeletal abnormalities such as fibro-osseous lesions of the jawbones, bone fragility, bowing of the lower limbs, and frequent severe fractures at early onset^3–5^.

Heterozygous mutations in the *ANO5* gene are by far the most common cause of GDD^6–9^. In addition, homozygous mutations in *ANO5* are also associated with limb-girdle muscular dystrophy type 2L (LGMD2 L) and Miyoshi-like distal myopathy (MMD3)^10–12^. TMEM16E is highly expressed in bone and skeletal muscle, suggesting its important role in the musculoskeletal system^6,7^. Concerning this issue, few *Ano5* gene editing animal models have been applied to investigate and dissect the function of *Ano5* in skeletal and muscular disease^13–17^. Although some aspects of the muscular phenotype were successfully displayed in *Ano5*-deficient mice and rabbits^13,15^, recapitulation of the stable skeletal phenotype has encountered some obstacles and difficulties^14,16^. Specifically, a study showed that marked increases in bone mineral density (BMD) and bone matrix mineralization were observed in *Ano5* knockout (KO) mice^15^; however, a similar skeletal phenotype could not be produced in another study^16^.

TMEM16E, encoding 913 amino acids, specifically localized in the endoplasmic reticulum (ER), is a member of the TMEM16/anoctamin protein family with 10 membrane-spanning helices. These proteins are involved in a variety of functions, including ion transients (Ca^2+^-activated Cl channels, CaCCs), calcium-dependent phospholipid scrambling (PLS), and regulation of other membrane proteins^18–24^. It was proposed that TMEM16E plays a role in muscle repair through a mechanism involving Ca^2+^ transients at the ER and annexins^25,26^ or Ca^2+^-activated phospholipid scramblases (PLSases)^27,28^. However, GDD‐related mutations are postulated to confer constitutive scrambling activity without the requirement of elevated cytosolic calcium^29^, so the exact molecular function of TMEM16E-regulated Ca^2+^ transients in bone homeostasis remains elusive.

Here, we report a de novo pathogenic *ANO5* frameshift insertion mutation in a GDD family. We also show that loss-of-function (LOF) of TMEM16E is associated with decreased mandibular and trabecular bone volume due to reduced osteoblast number, maturation, and bone-forming activity, as well as more significantly reduced osteoclast number and activity, in humans and mice. Moreover, TMEM16E is a regulator of Ca^2+^ transients via ER membrane-mediated Ca^2+^ signaling pathways, which are critical for WNT/β-Catenin activation for proper osteoblast generation and receptor activator of NF-κB ligand (RANKL)-induced nuclear factor of activated T cells c1 (NFATc1) activation of osteoclast generation. Accordingly, osteoanabolic treatment, such as parathyroid hormone (PTH) supplementation, can be used to ameliorate skeletal metabolism disorders in GDD patients. Overall, our data strongly suggest that *Ano5* constitutes a target for the treatment of bone homeostasis disorders.

## Results

### Patients with GDD and osteopenia carrying *ANO5* insertion mutations present loss of TMEM16E

**Fig. 1:**
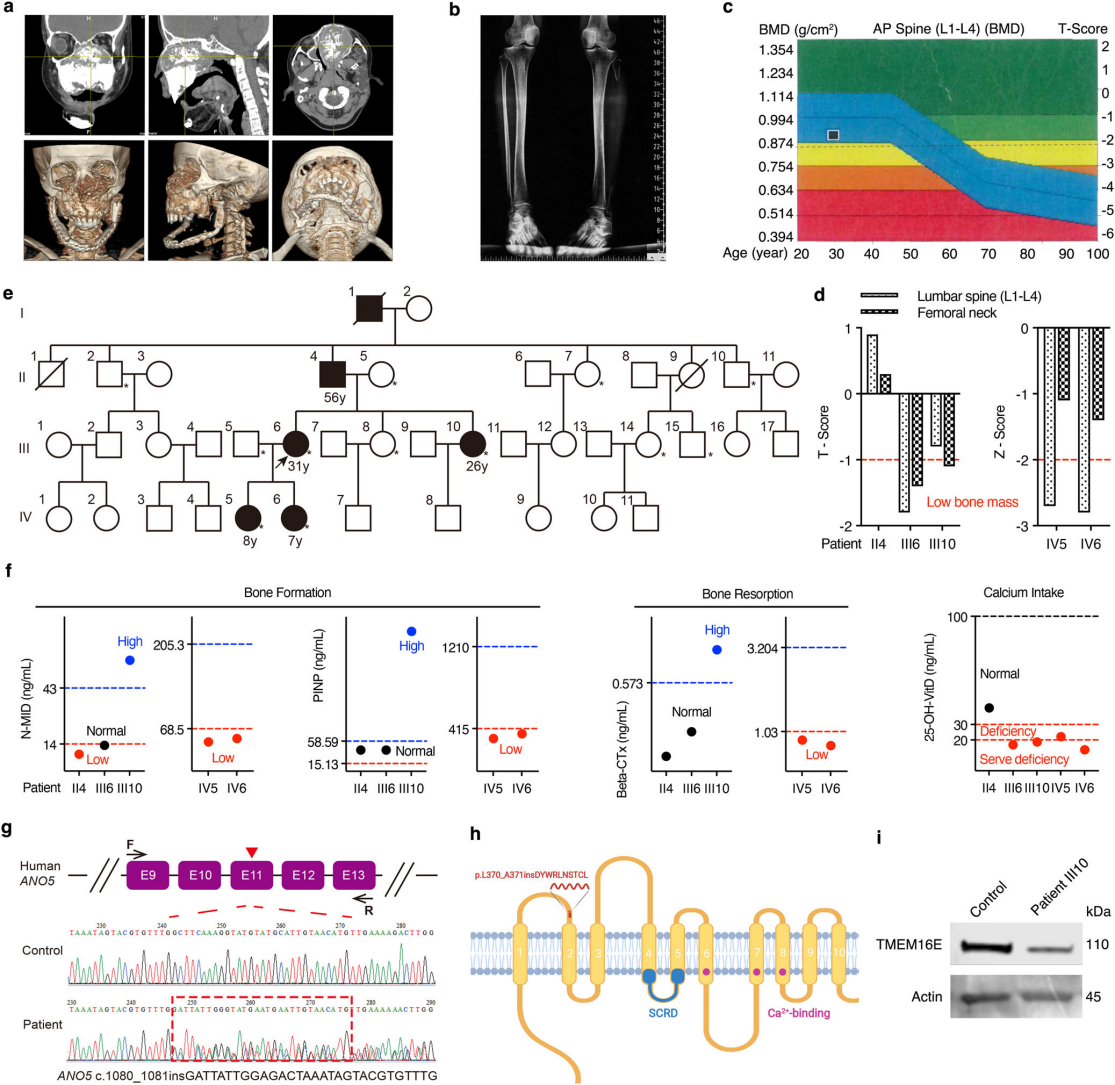
Patients with GDD and osteopenia carrying ANO5 insertion mutations present loss of TMEM16E. **a** 3D reconstruction image of the maxillofacial CT of the proband (III6). **b** Radiographs of the lower limbs of the proband (III6). **c** DXA showed low BMD at lumbar spine L1–L4 of the proband (III6). The clinical examination images were original from Shanghai Ninth Hospital Rare Diseases Database. **d** Low *T*-scores and *Z* scores were assessed by DXA in all affected patients. **e** Pedigree demonstrating the AD inheritance pattern of GDD. Generation, I–IV; males, squares; females, circles; patients, shaded black squares or circles; the proband (III6), an arrow; extracted DNA samples, an asterisk; age is labeled below. **f** Bone formation markers, bone resorption markers, and calcium intake markers of all patients were determined. **g** Schematic representation of the frameshift insertion on *ANO5* exon 11 (top) and Sanger sequencing validation of the frameshift in patient RNA-coding sequences compared with control sequences (bottom). Created based on human cDNAs sequence for *ANO5* (NCBI; GenBank accession no. NM_213599.3). **h** Schematic representation of the TMEM16E protein, which includes 10 TMDs, an SCRD, and three calcium-binding sites. The insertion site is indicated as a red line in the first extracellular loop. Created based on the human ANO5 (TMEM16E) sequence (Swiss Prot ID: Q75V66) with BioRender.com. **i** Representative images showing TMEM16E expression in PBMCs by Western blotting of samples from controls and patients.

A 22‐year‐old female was recruited at our hospital for recurrence of a large benign tumor in her oral cavity from 6 years old. Gradual and incessant enlargement involving the entire corpus of the maxilla caused severe facial disfigurement, and diaphyseal cortical thickening and mild bowing of tubular bones were also observed (Fig. 1a, b, Supplementary Fig. 1 a, b, III6). The patient sought to treat the maxillary mass and improve the mandibular deformity caused by the last operation 10 years ago. Therefore, we performed surgical treatment, and the histology of the jawbone lesions showed fibroblastic tissue proliferation intermixed with predominantly “cementum-like deposits” (Supplementary Fig. 1d, III6). We observed that similar skeletal abnormalities also occurred in other family members (Supplementary Fig. 1a–d). Moreover, the dual-energy X-ray absorptiometry (DXA) scan revealed low BMD with low *T* and *Z* scores in the lumbar spine L1–L4 and right femoral neck in patients and even showed osteoporosis in the proband’s two daughters (IV5, IV6) (Fig. 1 c, d). Laboratory serum tests further revealed low levels of the bone formation markers N-terminal/midregion (N-MID), procollagen type I N-terminal propeptide (P1NP), and bone resorption marker beta isomer of C-terminal telopeptide of type I collagen (beta-CTx) in young patients IV5 and IV6, and a severe deficiency in the calcium uptake marker 25‐hydroxyvitamin D (25-OH-VitD) in most patients (Fig. 1f). The detailed clinical findings of the probands and other affected patients are provided in Table 1 and Supplementary Fig. 1. The grandfather of the proband passed away before we established connection with him, and he also suffered from slightly swollen jawbones without any surgical treatment, according to family members. When hereditary, histologic and laboratory features were taken into consideration with clinical and radiographic features, GDD was the most appropriate diagnosis.

**Table 1:** Clinical findings and treatments of gnathodiaphyseal dysplasia patients.

Patient	Patient ID	II4	III6	III10	IV5	IV6
	Sex/Age (yr)	M/56	F/31	F/26	F/8	F/7
Body measurement	Height (cm)/Weight (kg)	165/57	151/39	153/40	113/20	112/20
Onset	Age at onset (yr)	11	6	18	7	7
	Initial symptoms	Mandibular and maxillary mass	Mandibular mass	Impacted tooth	Impacted tooth	NA
Features of jawbones	Gnathic location	Mandible and maxilla	Mandible and maxilla	Mandible and maxilla	Mandible	No
	Tooth	Some teeth missing, multiple impacted teeth in the inferior margin of the mandible	Multiple impacted teeth in the nasal side of the maxillary sinus and the inferior margin of the mandible	Some teeth missing, multiple impacted teeth in the nasal side of the maxillary sinus and the inferior margin of the mandible	Some teeth missing, multiple impacted teeth in the inferior margin of the mandible	No
	Typical radiologic features	Skin and plate repaired in the operation maxilla and mandible	Skin and plate repaired in the operation mandible, multifocal/multiquadrant progressively expansive maxilla with the surface convex and uneven; huge soft texture density masses adjacent to bilateral nasal cavities, left superior sinus, ethmoid, and left intraorbital muscles, and high-density patches scattered inside; the left eyeball compressed and anterior and laterally shifted	Patchy radiodense lesions adjacent to dental roots in maxilla and mandible; the jaws swelled slightly to the periphery; less dense shadows seen on the buccal side of left mandibular angle	Extensive radiopaque areas throughout the maxilla and the mandible	NA
Features of long bones		No	Cortical bone thickening	Cortical bone thickening	NA	NA
Treatments		Total maxillectomy and mandibulectomy, mandibular reconstruction using a titanium plate	Total mandibulectomy, mandibular reconstruction using a titanium plate (11 yr); mandibular reconstruction with free vascularized fibular graft, repaired by pectoralis major myocutaneous flap; subtotal bilateral maxillectomy (22 yr)	Removal of impacted mandibular and maxillary tooth	No	No
Histology of jawbone lesions		NA	Ossifying fibroma, a fibroblastic stroma with variable cellularity and a heterogeneous osseous component composed of woven bone and more cementum-like material	Florid cemento-osseous dysplasia, lesions near the apex of the teeth, and multifocal and multiquadrant involvement of tooth-bearing areas of the jaws	No	NA

*M* male, *F* female, yr years old, NA not available.

We illustrated the distribution of affected members in four generations of this family (Fig. 1e) and selectively performed whole-exome sequencing (WES) on 4 patients (II4, III6, III10, IV5) and 4 controls (II5, II10, III5, III8). After filtering, 9 gene variants remained (Supplementary Table 1, see the section “Methods”). Sanger sequencing of 1 other patient (IV6) and 4 other controls (II2, II7, III14, III15) confirmed a heterozygous *ANO5* frameshift insertion mutation (c.1080_1081insGATTATTGGAGACTAAATAGTACGTGTTTG, p.L370_A371insDYWRLNSTCL) (NM_213599.3) in exon 11 that was strongly predicted to be causative and a stop codon (Fig. 1g, Supplementary Table 2, see the section “Methods”). The TMEM16E insertion lies near the second transmembrane domain (TMD), which may cause the loss of predicted TMDs after the first TMD in the mutant TMEM16E protein (Fig. 1h). To assess the possible intracellular involvement of TMEM16E by using its antibody (aa 792–819), we studied TMEM16E expression in peripheral blood mononuclear cells (PBMCs) extracted from patients and controls. TMEM16E protein expression in patients was significantly lower in the GDD patients than in the controls (Fig. 1i). Taken together, these findings indicate that the heterozygous frameshift insertion mutation in exon 11 of the *ANO5* gene results in downregulated TMEM16E protein and the occurrence of GDD.

### *Ano5* deletion decreases bone formation and resorption, resulting in reduced bone volume in mice

**Fig. 2:**
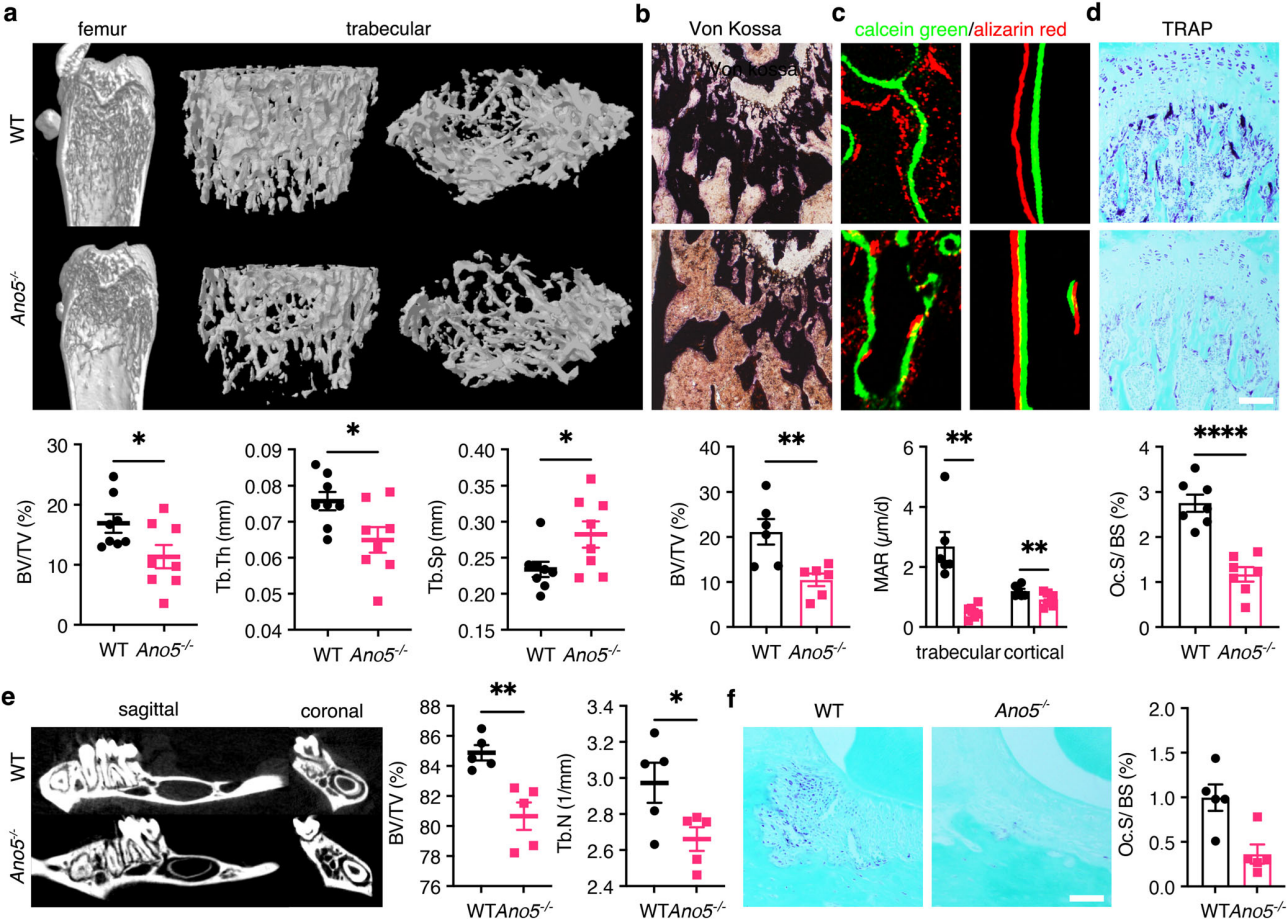
*Ano5* deletion decreases bone formation and resorption, resulting in reduced bone volume in mice. **a** µCT and 3D reconstruction of the proximal femur trabeculae of 8-week-old WT and *Ano5*^*−/−*^ mice, quantification of BV/TV, Tb. Th and Tb. Sp (*n* = 8). **b** Femur sections were stained with Von Kossa stain, and BV/TV was quantified (*n* = 6). **c** Dynamic histomorphometry of trabecular and cortical femurs and quantification of MAR (*n* = 6). **d** Femur sections with TRAP staining, quantification of Oc.S/BS (*n* = 7). **e** µCT of sagittal and coronal sections of mandibles from 8-week-old WT and *Ano5*^*−/−*^ male mice, quantification of BV/TV and Tb. N (*n* = 5). **f** Mandibular sections with TRAP staining, quantification of Oc.S/BS (*n* = 5). Scale bar for sections, 100 µm. Data are represented as the mean ± SEM. Two-tailed Student’s *t* test (for **a**, **b**, **d**–**f**) and two-way ANOVA (for **c**) followed by Tukey’s multiple comparisons test. *P < 0.05, **P < 0.01, ****P < 0.0001.

To substantiate the role of the loss of TMEM16E as a trigger of the GDD phenotype, we used a well-established *Ano5* KO mouse model^17^ and age- and sex-matched C57BL6/J mice as wild-type (WT) controls. Genotyping RT–PCR analysis revealed the presence of the KO allele in the heterozygous and homozygous mice, as expected, and the WT allele was not detected in the homozygous mice (Supplementary Fig. 2a). Four weeks after birth, bone microstructure μCT imaging of heterozygous *Ano5*^*+/−*^ and homozygous *Ano5*^*−/−*^mice revealed modest decreases in bone volume in both the femur and mandible, characterized by lower bone indices, including bone volume per tissue volume (BV/TV) and BMD (Supplementary Fig. 2b, c). *Ano5*^*+/*−^mice were phenotypically similar to *Ano5*^*−/−*^ mice and exhibited GDD-like skeletal phenotypes. To further identify changes in bone remodeling at an age similar to that of onset in humans, we analyzed 8-week-old *Ano5*^*−/−*^ homozygous mice. μCT analysis of the femur revealed that the femoral tubercular BV/TV was 23% lower, the trabecular thickness (Tb. Th) was 14% lower, and the trabecular separation (Tb. Sp) was 1.2-fold higher in *Ano5*^*−/−*^ mice than in control mice (Fig. 2a). In cortical bone, bone indices, including BV/TV, percent cross-sectional bone area (B.Ar/T. Ar), and percent cross-sectional bone perimeter (B. Pm/T. Pm) were not different between the groups (Supplementary Fig. 2d). The low bone volume phenotype was also reproduced in the mandible: the BV/TV was 34% lower, and the Tb. N was 10% lower in *Ano5*^*−/−*^ mice than in controls (Fig. 2e).

Bone sections with Von Kossa staining also showed decreased trabecular bone surface area in *Ano5*^*−/−*^ versus WT mice (Fig. 2b). To elucidate the involved mechanisms, we assessed osteoblast and osteoclast activity status in bone sections by analyzing dynamic bone formation and tartrate-resistant acid phosphatase (TRAP). Bone sections subjected to calcein green/alizarin red (AR) double labeling showed a decreased mineral apposition rate (MAR) in *Ano5*^*−/−*^ versus WT mice (Fig. 2c). Concerning osteoclast status, TRAP-stained bone sections exhibited a reduction in osteoclast numbers and osteoclast surface size in *Ano5*^*−/−*^ versus WT mice (Fig. 2d, f). These data suggest that the absence of *Ano5* results in an intrinsic defect in bone formation and resorption in vivo.

### *Ano5* positively regulates osteoblast and osteoclast differentiation

**Fig. 3:**
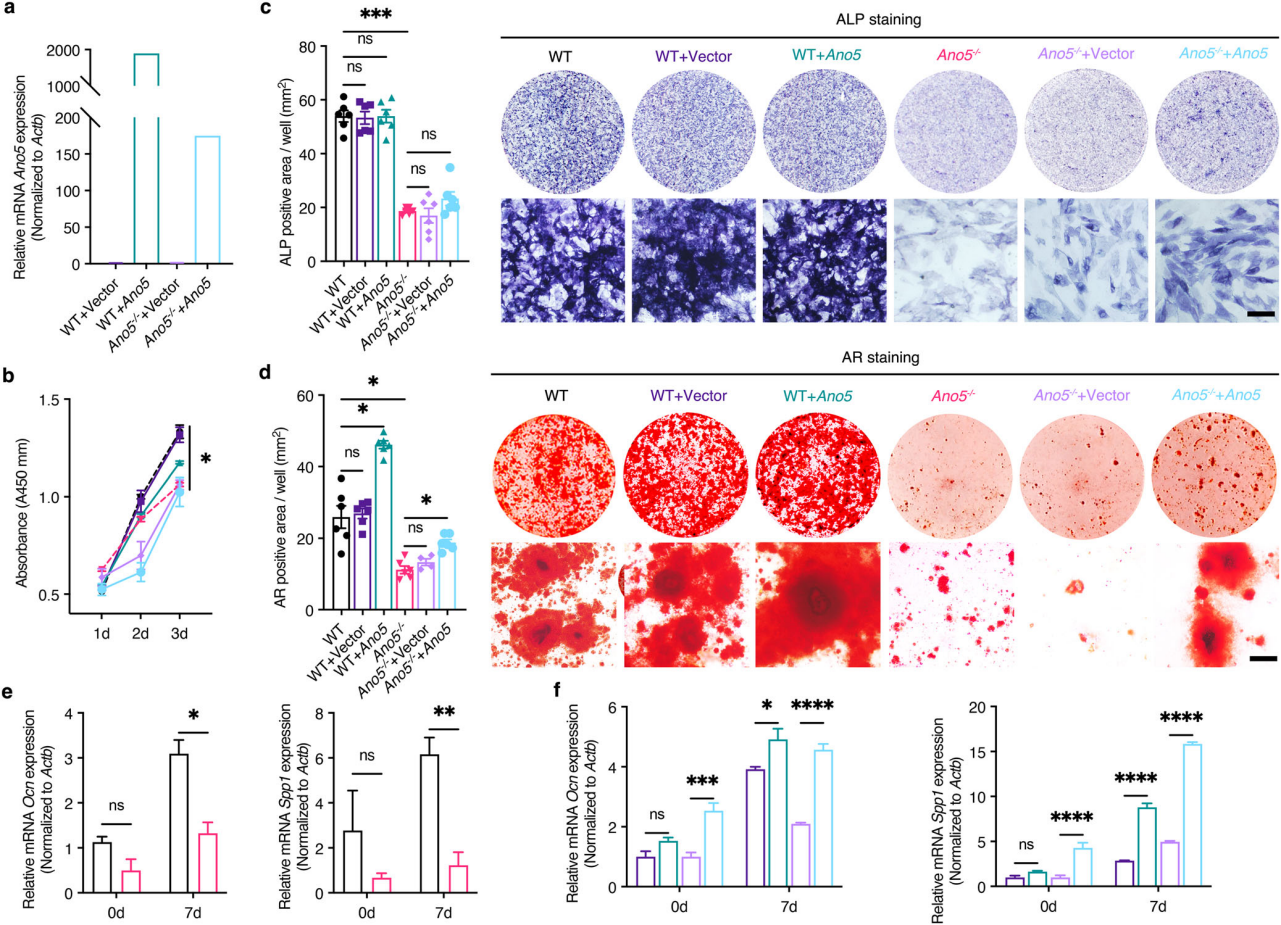
*Ano5* positively regulates osteoblast differentiation. **a** Overexpression of *Ano5* in cells transduced with lentivirus was confirmed by qPCR. **b** The proliferation of WT and *Ano5*^*−/−*^ calvarial osteoblasts and those transduced with a control vector or *Ano5* overexpression lentivirus by CCK-8 assay. **c** Representative images and quantification of ALP staining after 7 days of osteogenic differentiation. **d** Representative images and quantification of AR staining after 21 days of osteogenic differentiation. **e** Relative mRNA expression of *Ocn* and *Spp1* in osteoblasts differentiated from WT and *Ano5*^*−/−*^ calvarial osteoblasts and **f** those transduced with a control vector or *Ano5* overexpression lentivirus as a function of time in culture. Scale bar, 50 µm. All results are representative of data generated from at least three independent experiments. Data are represented as the mean ± SEM. Two-way ANOVA followed by Tukey’s multiple comparisons test. *P < 0.05, **P < 0.01, ***P < 0.001, ****P < 0.0001; ns: not significant *P* > 0.05.

To determine the cellular basis for the role of *Ano5* in osteoblast generation, we compared the differentiation capacity and bone-forming potential of primary calvarial osteoblasts and bone marrow mesenchymal stem cells (BMSCs) from WT and *Ano5*^*−/−*^ mice. Additionally, to assess whether the alterations detected in *Ano5*-deficient osteoblasts were attributable to *Ano5* compensatory mechanisms, we re-expressed *Ano5* in WT and *Ano5*^*−/−*^ calvarial osteoblasts by administration of adenoviruses encoding either *Ano5* or control vector (Fig. 3a). Cell proliferation rates were lower in *Ano5*^*−/−*^ than WT osteoblasts over a 7-day culture period, and they remained unchanged upon *Ano5* re-expression (Fig. 3b, Supplementary Fig. 3a). *Ano5*^*−/−*^ osteoblast cultures showed reduced alkaline phosphatase (ALP) activity (Fig. 3c, Supplementary Fig. 3b, d) and significantly fewer bone nodules than WT cultures after 3 weeks in differentiation media (Fig. 3d, Supplementary Fig. 3c, d). After *Ano5* re-expression, ALP activity was slightly increased in *Ano5*^*−/−*^ osteoblasts, and the mineral nodule formation ability was obviously improved in WT osteoblasts and partly improved in *Ano5*^*−/−*^ osteoblasts (Fig. 3c, d). Likewise, decreased differentiation potential was confirmed by reduced mRNA expression of osteocalcin (*Ocn*) and secreted phosphoprotein 1 (*Spp1*) in differentiating *Ano5*^*−/−*^ osteoblasts (Fig. 3e), and the expression of those genes was markedly upregulated after *Ano5* re-expression in *Ano5*^*−/−*^ and WT osteoblasts (Fig. 3f). Thus, similar to their counterparts in vivo, *Ano5*^*−/−*^ osteoblasts differentiated poorly and showed functional defects in matrix deposition and bone formation.

**Fig. 4:**
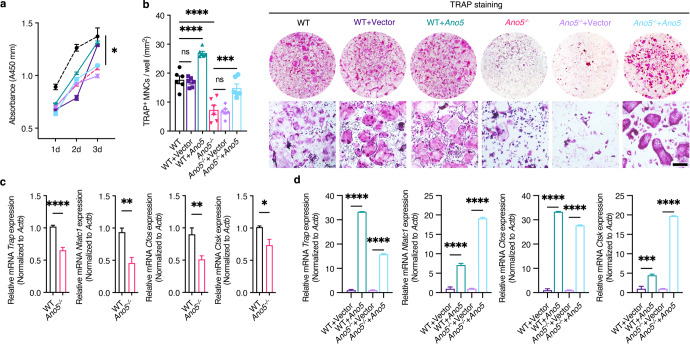
*Ano5* positively regulates osteoclast differentiation. **a** The proliferation of WT, *Ano5*^*−/−*^ BMMs, and those transduced with a control vector or *Ano5* overexpression lentivirus by CCK-8 assay. **b** Representative images and quantification of TRAP staining after 5–7 days of osteoclast differentiation. Scale bar, 50 µm. **c** Relative mRNA expression of *Trap, Nfatc1, Cfos,* and *Ctsk* in osteoclasts differentiated from WT, *Ano5*^*−/−*^ BMMs and **d** those transduced with a control vector or *Ano5* overexpression lentivirus. All results are representative of data generated from at least three independent experiments. Data are represented as the mean ± SEM. Two-tailed Student’s *t* test (for **c**) and two-way ANOVA followed by Tukey’s multiple comparisons test (for **a**, **b**, **d**). *P < 0.05, **P < 0.01, ***P < 0.001, ****P < 0.0001; ns: not significant *P* > 0.05.

To determine the intrinsic cellular role of *Ano5* in osteoclast generation, we compared the proliferation rates of *Ano5*^*−/−*^ and WT bone marrow-derived macrophages (BMMs) and those after *Ano5* re-expression in vitro (Fig. 4a). When purified BMMs were induced to become mature osteoclasts with RANKL, significantly fewer TRAP+ multinucleated cells (MNCs) were observed in *Ano5*^*−/−*^ BMMs than in WT BMMs, and they returned to control levels after re-expressing *Ano5* in *Ano5*^*−/−*^ BMMs (Fig. 4b). We also revealed that the levels of *Trap*, *Nfatc1*, *c-Fos*, and cathepsin K (*Ctsk*), which are markers of differentiated osteoclasts, were significantly lower (Fig. 4c) but markedly upregulated after *Ano5* re-expression in *Ano5*^*−/−*^ cells (Fig. 4d). These results suggest that *Ano5* is a crucial regulator of osteoclast differentiation, as its absence reduced the number of mature osteoclasts. Overall, *Ano5* is directly responsible for the cellular functions of osteoblasts and osteoclasts, including differentiation, bone remodeling, and gene transcription.

### LOF of TMEM16E impairs intracellular calcium oscillations

**Fig. 5:**
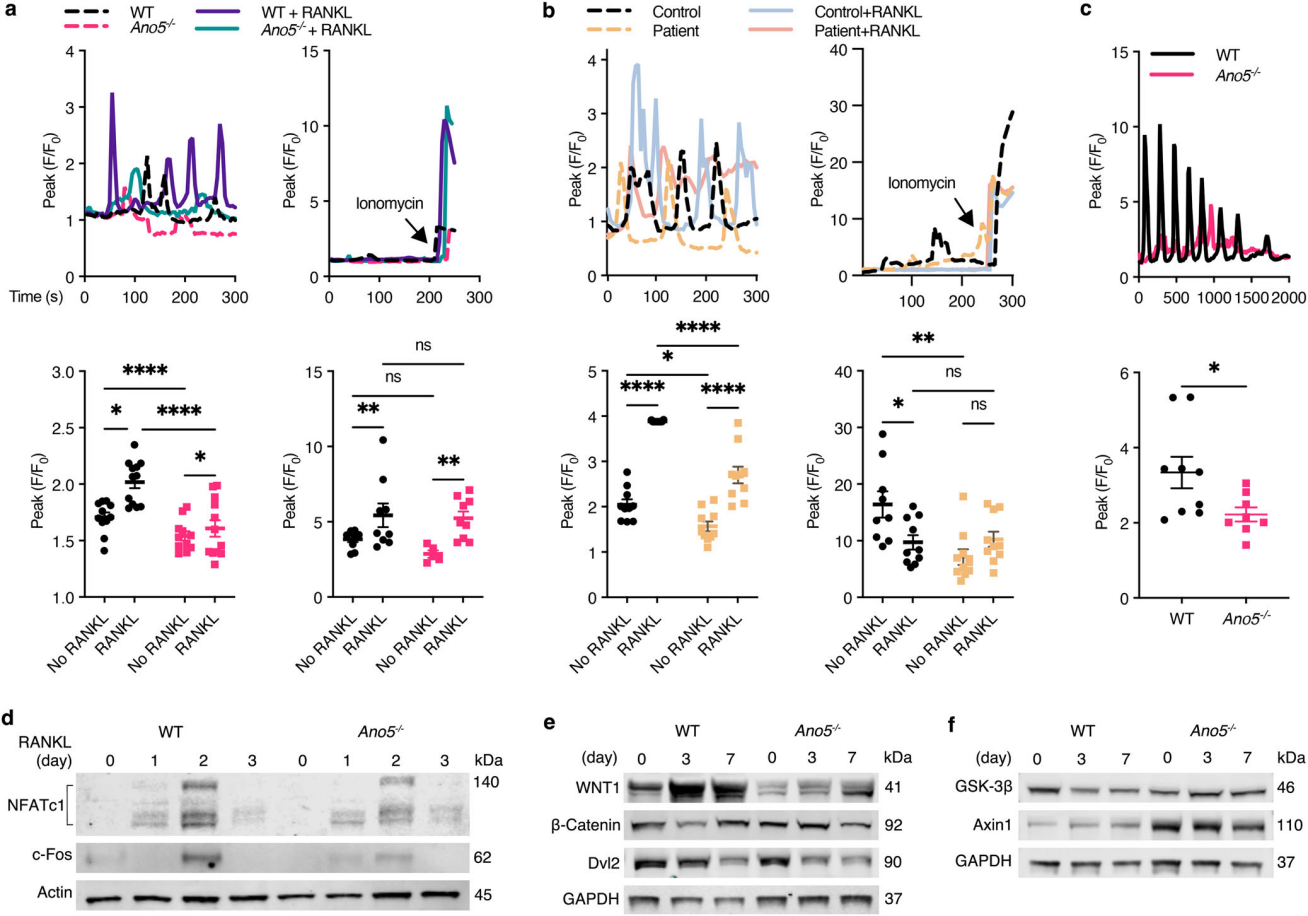
LOF of TMEM16E impairs intracellular calcium oscillations. **a** RANKL-induced oscillatory changes in [Ca^2+^]_i_ from *Ano5*^*−/−*^ and WT BMMs (left) and after the addition of 10 mM ionomycin at the end of each experiment (right). **b** RANKL-induced oscillatory changes in [Ca^2+^]_i_ in PBMCs from patients and healthy controls (left) and after the addition of 10 mM ionomycin at the end of each experiment (right). **c** Oscillatory changes in [Ca^2+^]_i_ during osteogenic differentiation of *Ano5*^*−/−*^ and WT calvarial osteoblasts. **d** NFATc1 and c-Fos expression as assessed by Western blotting of WT and *Ano5*^*−/−*^ BMM nuclear samples during osteoclast differentiation. **e** WNT1, β-Catenin, Dvl2, **f** GSK-3β, Axin1 expression as assessed by Western blotting of osteoblast samples from WT and *Ano5*^*−/−*^ mice during osteoblast differentiation. Fluorescence intensity values over time (normalized to minimum intensity values) were calculated for each cell. Analysis of the peak fluorescence intensity per firing cell over an observation period. Data are represented as the mean ± SEM. All results are representative of data generated from at least three independent experiments. Two-tailed Student’s *t*-test (for **c**), two-way ANOVA (for **a**, **b**) followed by Tukey’s multiple comparisons test. *P < 0.05, **P < 0.01, ****P < 0.0001; ns: not significant *P* > 0.05.

To assess whether TMEM16E may regulate Ca^2+^ homeostasis in bone metabolism by regulating intracellular Ca^2+^ oscillations, we analyzed the dynamic [Ca^2+^]_i_ changes in *Ano5*^*−/−*^ BMM-induced osteoclasts. We showed that RANKL-induced [Ca^2+^]_i_ slowly increased and returned to baseline in *Ano5*^*−/−*^ osteoclasts compared with WT osteoclasts, whereas no changes were observed for BMMs cultured without RANKL (Fig. 5a left). After stimulation with ionomycin, the average number of peaks of [Ca^2+^]_i_ oscillations was dramatically enhanced in *Ano5*^*−/−*^ osteoclasts and remained the same in WT osteoclasts (Fig. 5a right). We next studied TMEM16E*-*mediated Ca^2+^ changes in osteoclasts cultured from PBMCs in GDD patients and normal controls. We did observe few differences in the [Ca^2+^]_i_ oscillations between normal control macrophages and GDD patient macrophages (Fig. 5b left). Furthermore, we examined RANKL-induced [Ca^2+^]_i_ oscillations, which were impaired in patients’ osteoclasts, exhibiting much lower and slower returns to baseline levels (Fig. 5b left). After being induced by ionomycin, Ca^2+^ transients were significantly elevated in both groups (Fig. 5b right). To investigate RANKL-dependent signaling pathways and the calcium-NFATc1 pathway driving osteoclastogenesis, we performed Western blotting on RANKL-treated BMMs. After long-term stimulation with RANKL, the nuclear expression levels of c-Fos and NFATc1 were decreased in *Ano5*^*−/−*^ BMMs compared with WT BMMs (Fig. 5d). These data suggest that LOF of TMEM16E can block RANKL-induced c-Fos and NFATc protein translocation into the cell nucleus.

Given the role of *Ano5* in matrix deposition and bone formation, we explored whether [Ca^2+^]_i_ oscillations might be changed in *Ano5*^*−/−*^ osteoblasts. Consistent with the results in osteoclasts, sustained [Ca^2+^]_i_ oscillations were observed in WT cells; however, [Ca^2+^]_i_ oscillations were abolished in osteoblasts from *Ano5*^*−/−*^ mice (Fig. 5c). To investigate the calcium-related WNT/β-Catenin pathway during ossification, we performed immunoblotting of total proteins from osteoblasts with antibodies specific for WNT1, β-Catenin, disheveled homolog (Dvl2) and its negative regulator's glycogen synthase kinase 3 beta (GSK-3β) and Axin1. The expression of WNT1 was highly downregulated during differentiation in *Ano5*^*−/−*^ osteoblasts compared with that in WT osteoblasts, and the expression of β-Catenin and Dvl2 was also downregulated in *Ano5*^*−/−*^ osteoblasts compared with WT osteoblasts after 7 days of differentiation (Fig. 5e), whereas the expression of the negative regulators GSK-3β and Ainx1 was upregulated in *Ano5*^*−/−*^ and WT osteoblasts (Fig. 5f). Thus, modulation of the WNT/β-Catenin pathway by TMEM16E is necessary for subsequent calcium uptake-induced osteoblast mineralization. Taken together, these data strongly suggest that [Ca^2+^]_i_ oscillations are substantially decreased in *Ano5*^*−/−*^ osteoblasts and osteoclasts, resulting in a reduction in WNT/β-Catenin signaling and NFATc1 activation, which suggests that TMEM16E might function as an intercellular Ca^2+^-permeable channel in bone metabolism.

### PTH enhances bone strength in osteopenia

**Fig. 6:**
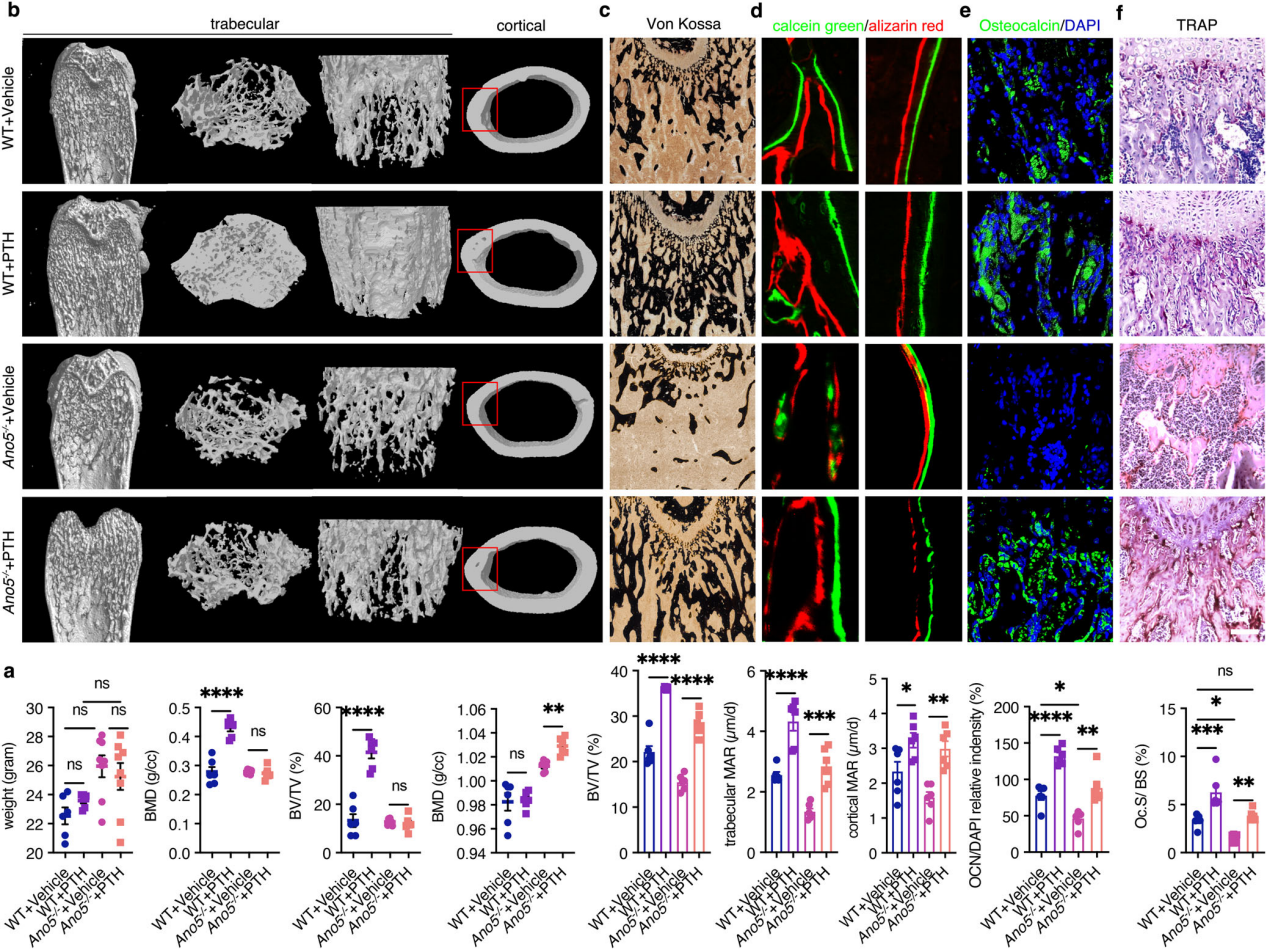
PTH enhances bone strength in osteopenia. **a** Growth weight of 8-week-old WT and *Ano5*^*−/−*^ male mice after PTH treatment and vehicle treatment. **b** μCT and 3D reconstruction of the trabecular and cortical femurs of 5-week-old WT and *Ano5*^*−/−*^ male mice after 3 weeks of PTH and vehicle treatment, and quantification of BMD and BV/TV. **c** Femur sections were stained with Von Kossa stain, and BV/TV was quantified. **d** Dynamic histomorphometry of trabecular and cortical femurs, quantification of MAR. **e** Femur sections were stained with OCN immunohistochemistry for quantification of relative OCN intensity. **f** Femur sections were stained with TRAP stain and quantified by Oc.S/BS. *N* = 6 for each group. Scale bar for sections, 100 µm. Data are represented as the mean ± SEM. Two-way ANOVA was followed by Tukey’s multiple comparisons test. *P < 0.05, **P < 0.01, ***P < 0.001, ****P < 0.0001; ns: not significant *P* > 0.05.

To find treatment to increase bone density and prevent long bone fractures in GDD patients, we next evaluated the therapeutic potential of the osteoanabolic medicine PTH in osteopenic *Ano5*^*−/−*^ and WT mice. After 3 weeks of treatment, there was no effect on the body weight in the four groups (Fig. 6a). PTH achieves an outstanding therapeutic effect in femoral trabecular bone in PTH-treated WT mice, while it exhibits a significant therapeutic effect in femoral cortical bone in *Ano5*^*−/−*^ mice. μCT analysis showed that BMD and BV/TV of trabecular femurs were significantly higher in PTH-treated WT mice, while BMD of cortical femurs was typically higher in PTH-treated *Ano5*^*−/−*^ mice (Fig. 6b). After PTH treatment in both *Ano5*^*−/−*^ and WT mice compared to vehicle-treated controls, histomorphometric Von Kossa staining analysis of the BV/TV ratio was increased (Fig. 6c); double-labeled bone sections also showed obviously increased MAR (Fig. 6d); the number of mature OCN-expressing osteoblasts was higher (Fig. 6e); and the number of TRAP+ matured osteoclasts was higher (Fig. 6f). Overall, our data strongly suggest that PTH, as a calcium intake mediator, partly improved the bone strength and structural characteristics of the femur in the *Ano5* loss mouse model.

## Discussion

GDD is a rare hereditary systemic skeletal syndrome that includes the maxillofacial bone along with the rest of the bones. To achieve a radical cure and prevent the recurrence of maxillofacial disorders, appropriate surgical management and facial reconstruction are needed in GDD patients^5,30,31^. However, GDD abnormalities in long bones, the ilium, the skull, and bone mass density lack effective treatments and are also the key factors that distinguish it from common genetic maxillofacial diseases^4,32–34^. Although most patients suffer from similar thickening of the diaphyseal cortex of the femur and frequent fractures, which affect body weight support and daily movement, GDD patients with different *ANO5* mutations show diverse phenotypes. Patients with *ANO5* p.L370_A371ins mutation in our study showed decreased trabecular dense bony deposits associated with low-bone-mass disorder^4,8,32^, while those with *ANO5* p.Ser500Phe mutation showed increased high bone turnover pathology accompanied by high-bone-mass disorder^9,35^. Additionally, the mechanisms by which different *ANO5* mutations contribute to aberrant bone homeostasis remain unclear. In that case, the identification of *ANO5* mediating bone homeostasis will facilitate the development of new therapies and may improve responses to currently available therapies. In this study, by measuring genotypes and phenotypes in a GDD family with low bone mass osteopenia, we report a de novo pathogenic *ANO5* frameshift insertion mutation (c.1080_1081insGATTATTGGAGACTAAATAGTACGTGTTTG, p.L370_A371insDYWRLNSTCL). Our results also confirm that the mutation resulted in partial instead of complete LOF of the TMEM16E protein. First, our GDD family with the mutation did not have a history of severe bone fractures, although it presented decreased BMD throughout the body. Besides, we show that the phenotypes of bone loss in heterozygous *Ano5*^*+/−*^ mice are similar to but less severe than those in homozygous *Ano5*^*−/−*^ mice. Moreover, the introduction of *Ano5* into *Ano5*^*−/−*^ primary osteoblasts and BMMs was sufficient to rescue osteogenesis and osteoclastogenesis. Thus, our results suggest that *ANO5* is dosage-sensitive and that LOF of the TMEM16E protein caused by the heterozygous *ANO5* mutation in one allele is partly compensated by another normally functional allele.

The [Ca^2+^]_i_ concentration in bone cells is high, and maintenance of its concentration and external Ca^2+^ balance is crucial for bone homeostasis. Recent studies have highlighted the importance of intracellular Ca^2+^ signaling for osteoclast differentiation^36–38^. There are two sources of intracellular calcium in cells: inflow from the extracellular space and release from intracellular stores, such as the ER^39^. Notably, Ca^2+^-mobilized from ER stores increases the intracellular Ca^2+^ concentration and calcium oscillations and then activates NFATc1, which translocates to the nucleus and induces osteoclast-specific gene transcription, promoting osteoclast differentiation^40,41^. On the other hand, RANKL also evokes calcium oscillations, leading to the activation of NFATc1^36^. Moreover, intercellular communication between osteoclasts and osteoblasts through calcium oscillations contributes to efficient osteoclastogenesis in vivo. Recent data have highlighted the WNT/β-Catenin pathway as a regulator of bone mass that mediates multiple mechanisms, including the renewal of stem cells, stimulation of primary osteoblast replication, induction of osteoblastogenesis, and inhibition of osteoblast and osteocyte apoptosis^2,42,43^. These reports are consistent with our finding that LOF of TMEM16E inhibits [Ca^2+^]_i_ oscillations and inactivates the WNT/β-Catenin and RANKL–NFATc1 pathways decreasing osteogenesis gene translation and calcium deposition by osteoblasts and impairing osteoclastogenesis gene translation, osteoclast differentiation, and activity. Furthermore, inhabited *Ano5*^*−/−*^ osteoblast maturation also results in decreased stimulation of RANKL, greatly impeding the differentiation of osteoclasts from BMMs. In those ways, a more dramatic impaired osteoclast phenotype was found in *Ano5*^*−/−*^ mice with significantly reduced bone resorption, leading to disruption of the homeostasis of bone formation. When old osseous tissue cannot be absorbed by osteoclasts, signals are unable to be sent to osteoblasts to generate new osseous tissue, bringing about a decrease in bone mass and degenerated osteoclast resorption activity and thus leading to a vicious cycle of bone homeostasis. Based on these data, our study also sheds light on how bone homeostasis, which involves the balance of bone formation and resorption, can be changed to induce skeletal abnormalities in humans and mice when *ANO5* function is lost.

Meanwhile, Zanni et al. found that some GDD-related amino acid substitutions caused gain-of-function (GOF) of TMEM16E, which gives rise to constitutive phospholipid scrambling activity without the requirement for elevated cytosolic calcium, while MMD‐related amino acid substitutions have been identified to cause a loss of TMEM16E Ca^2+^‐activated phospholipid scramblase function^44^. In our study, we found that the PLS activity of *Ano5*^*−/−*^ osteoblasts and osteoclasts in Annexin V binding assays were similar to that of WT osteoblasts and osteoclasts either in the presence or absence of cytosolic Ca^2+^ (Supplementary Fig. 4). In that case, GOF of TMEM16E protein results in high-bone-mass phenotype GDD by hyperfunction of PLS with increased osteoblast and osteoclast indices, while LOF of TMEM16E protein causes low-bone-mass phenotype GDD by impaired function of calcium oscillations with decreased osteoblast and osteoclast indices. Overall, our results suggest the existence of GDD-sub-entities with high and low BMD phenotypes due to different GOF and LOF mutations and functions.

Recently, Jin, L. et al. noticed that expression levels of two mutant TMEM16E proteins (p.Cys356Tyr, p.Cys360Tyr) were much lower than those of wild-type TMEM16E, which is consistent with our finding of low TMEM16E expression with the LOF *ANO5* frameshift mutation^45^. In their study, the 73-year-old proband did not experience frequent fractures and exhibited thickening of the cortical long bones in the family with *ANO5* p.Cys360Tyr mutation, while the 15-year-old proband suffered repeated fractures in the family with *ANO5* p.Cys365Tyr mutation^45^. Our observations also showed a more obvious severe clinical phenotype and abnormal bone turnover markers in patients of young ages, especially in childhood and adolescence. Additionally, Li, H. et al., Kim, J. H. et al., and our study reached the same conclusion that *Ano5* significantly enhanced RANKL-induced osteoclast differentiation and bone resorption via multiple signal transduction processes^46,47^. Therefore, low bone resorption activities in elderly GDD patients explain why their bone mass is still maintained at normal or even increased levels when ordinary elderly people develop osteoporosis. Similarly, Wang, X. et al. and Jin, L. et al. found that *Ano5*-deficient and *Ano5*^*KI/KI*^ p.Cys360Tyr mutation mice showed increased BMD in cortical bones but reduced trabecular thickness in femurs and tibias by using 12- and 16-week-old mouse models as research objects^15,45^. As an age-related disease, the onset of GDD occurs at a very young age, mostly in juveniles between 10 and 16 years old; thus, by using 12-week-old and even 16-week-old mice as models of young adults and middle-aged humans, osteoporosis symptoms may be masked by aging changes in mice. The authors also noticed that calvaria-derived *Ano5*^*−/−*^ and *Ano5*^*KI/KI*^ osteoblasts show increased mineralization and osteoblastogenesis, which partly contradicts our conclusions^15,45^. According to our Von Kossa staining, OCN immunohistochemistry, and calcein/AR double-labeled sections of *Ano5*^*−/−*^ mouse femurs and osteoblast differentiation, *Ano5* strongly contributes to osteoblast maturation and bone formation. Based on the diversities in the development of the patient and mouse biological bone metabolic rhythm in different ages and skeletal locations, this observation may be due to the uneven distribution of calcium salt deposits depending on various times and spaces, so it is reasonable for the slight discrepancy to exist when generalizing the systemic osteogenesis situation, and different *ANO5* mutations contribute to aberrant bone homeostasis.

Another study showed that 12- and 24-week-old *Ano5* KO and knock-in (KI) mouse models harboring the p.Ter491Phe mutation represented no significant GDD-like phenotype with p.Ser500Phe mutation^16^. The ages of the mice used for analysis are also too advanced to successfully recapitulate GDD-like phenotypes. Moreover, the amino acid sequence is not conserved between humans and mice at that mutation site, where Ser is mutated to Phe in humans while Ter is mutated to Phe in mice. Because there are some differences between Ser and Ter in structure and biological function, it is possible that *Ano5* KI mice with p.T491F amino acid exchange did not display alterations in skeletal microarchitecture or mandible morphology. For example, threonine is an essential amino acid made up of an α-amino group, but serine is not. Serine plays an important role in catalytic function in many enzymes, such as trypsin, but threonine does not. Thus, we used 6- to 8-week-old mice as the “juvenile” range to reduce the likelihood that a late bloomer would develop during the experiment, and after 8 weeks of age, the mice were considered adults with all organs, like bone, maturely developed. By using 8-week-old *Ano5* KO mice for animal models, we simulated the low-bone-mass GDD phenotype with osteoporosis at the age of onset.

Our results also initially confirm that the *Ano5* gene expression is a necessary stage for PTH to promote bone homeostasis. After intermittent administration of PTH to WT and *Ano5*^*−/−*^ mice, structural parameters related to bone formation were markedly increased in PTH-treated WT mice, whereas in *Ano5*^*−/−*^ mice, although PTH partly treated them by increased cortical thickness and matrix mineralization, there were no significant differences compared to the WT groups. We concluded that the absence of *Ano5* might partially hinder PTH-driven bone anabolism and bone formation. For the osteoporosis-like phenotype GDD with low bone mass and low bone turnover, injectable PTH could be the best osteoanabolic treatment currently available for bone formation and activating WNT signaling in osteoblasts^48,49^. Additional calcium supplementation can also be used to prevent bone loss. For GDD with high bone mass and a high bone turnover phenotype, we can choose antiresorptive treatments, such as bisphosphonates and denosumab, which help to inhibit the differentiation and maturation of osteoclasts and reduce bone resorption. There are no established guidelines for the management of fracture risk in these patients. Physicians should assess the patient’s skeletal status more accurately, understand the mechanisms of drugs, and formulate a treatment plan under consideration of individual differences in patients.

Although some important discoveries were revealed by our studies, there are also limitations. The jawbone phenotypes of GDD, such as cemento-ossifying fibroma and impacted teeth, are generally regarded as calcified odontogenic tumors. Odontogenesis relies upon a series of reciprocal signaling interactions between the oral epithelium and adjacent neural crest-derived mesenchyme, the molecular mechanisms of which involve intracellular MAPK, Hedgehog, and Wnt pathways^50,51^. *ANO5* mutations must be related to abnormal odontogenic tumor development and/or to an epithelial–mesenchymal transition proposed to occur during normal root formation. According to our patients’ pathological results of jaw lesions, normal cancellous bones of the jaw were structurally destroyed and poorly mineralized, occupied by a large amount of fibrous tissue and cementum, leading to fibrous cementum-like lesions. We also identified decreased cancellous bone thickness and density in *Ano5*^*−/−*^ mouse mandibles, although some developmental odontogenic phenotypes were not observed. It is possible that some additional factors triggering the manifestation of certain aspects of the disorder are different in mice and humans, such as mandibular development, metabolism, or other biological characteristics. Multiple animal models have been used to study GDD but have not been met with complete success. To fully represent the characteristic pathologic changes observed in GDD, some large animal models could be used, such as horses, sheep, dogs, and pigs, which are arguably the most powerful models of human organ systems, including the jaw and other bones, and offer the advantages of anatomical, physiological, metabolic and genetic similarities with humans. Second, to fully address why GOF and LOF mutations both lead to GDD, a KI model with specific patient mutations would be more suitable to recapitulate GDD. In addition, cell-type-specific gene edits of *Ano5*, such as osteoblast, osteoclast, and odontogenic-like cell lines, still require future studies.

**Fig. 7:**
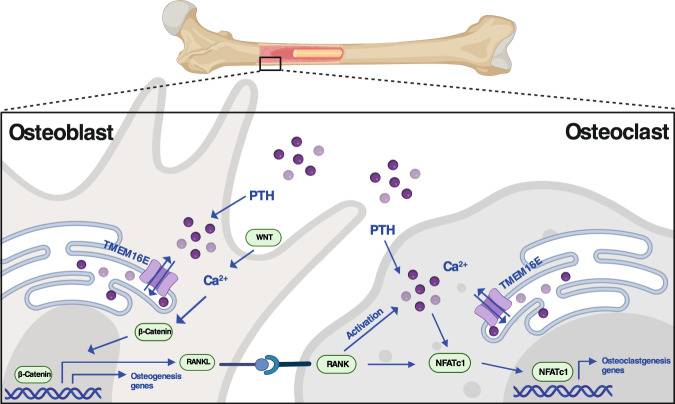
Schematic diagram of TMEM16E affecting bone metabolism through intracellular Ca^2+^ signal transmission. Shown are mechanisms by which TMEM16E in the ER leads to cytosolic Ca^2+^ spiking for activation of the WNT/β-Catenin signaling pathway and RANKL-stimulated NFATc1, thereby triggering Ca^2+^ signaling cascades that promote osteoblast and osteoclast differentiation and activation. Created with BioRender.com.

In summary, this study of individuals carrying the *ANO5* mutation p.L370_A371insDYWRLNSTCL improved our understanding of the causative pattern of LOF of TMEM16E in GDD families. Importantly, our data support the view that TMEM16E at the ER membrane of bone cells sustains Ca^2+^ transitions to the cytoplasm (Fig. 7). The effects on Ca^2+^ oscillations may explain the role of *Ano5* in the maintenance of bone homeostasis and related processes, such as matrix mineralization and osteoblast and osteoclast differentiation and function. Therefore, our research proposes *ANO5* as a therapeutic target for counteracting the development of skeletal diseases related to GDD.

## Methods

### Human subjects and clinical assessments

This research was supported by the Clinical Research Center, Scientific Research and Planning Department at Ninth People’s Hospital (SH9H), Shanghai Jiao Tong University School of Medicine (JYHJB01). The human samples used in this research project were obtained with evaluation and approval from the Institutional Ethics Committee of Ninth People’s Hospital (SH9H-2020-T99-3), and all ethical guidelines conformed to the 2008 Helsinki Declaration. The project was registered under the Ninth People’s Hospital Tissue Bank (C01). All patients (or the parents of patients) and family members provided written informed consent, and they also consented to have their photographs and clinical data used for publication. Five affected individuals and eight unaffected family members from the pedigree were included in this study. When participants were enrolled in the rare diseases research project, ancillary study procedures to measure aBMD, X-ray, and CT scans of the maxillofacial and lower extremities were performed. aBMD was measured by DXA using a Lunar iDXA X-ray bone densitometer system (GE Healthcare, Chicago, IL, USA). The aBMD, X-ray and CT scans were performed by qualified technologists and interpreted by radiologists who were not involved in the design or implementation of the study. Bone turnover markers in plasma were measured by the certified clinical diagnostic laboratory of Ninth People’s Hospital. There are no reference ranges for bone turnover markers in children from Ninth People’s Hospital, so we used the reference intervals recommended by the journal *Bone*^52^.

### Mice

This project has been assessed favorably by the Institutional Animal Care and Use Committee from Ninth People’s Hospital (JY-IACUC): the IACUC deemed that the abovementioned project complies with standard ethical regulations (SH9H-2020-A42-1). All animal work was approved and conducted according to ARRIVE guidelines. C57BL/6 mice were purchased from JieSiJie Laboratory Animal Corporation (Shanghai, China). *Ano5* KO mice (C57BL/6-Ano5<tm1Itak>) were obtained from RIKEN BioResource Center (Kyoto, Japan) and maintained at the Animal Resources Center at Ninth People’s Hospital under specific pathogen-free (SPF) conditions; they were backcrossed with C57BL/6J mice. The genomic DNA from KO mice was amplified by PCR using a forward primer (5′-GGTTGTATTGGTTCTTAAATTGTGG) and two reverse primers (5′-AACCGAAGACTGTCACATGTGGAAT for the 257 bp-WT allele and 5′-AATTCATTCTCGATTCTTGATGG for the 407 bp-mutant allele) as described. Both male and female mice were used in this study; age- and sex-matched 4- to 6-week-old mice were used for in vivo studies, and 6- to 8-week-old mice were used for in vitro studies. For PTH-treated *Ano5*^*−/−*^ mice, recombinant human PTH 1–34 (Beyotime, Shanghai, China) (80 µg/kg) and vehicle (NaCl) subcutaneous injections were administered 5 days per week over 3 weeks when the mice were 5–8 weeks old. Mice were maintained with a 12 h light/dark cycle and were fed standard chow. Cohoused animals were used for in vivo analyses. Littermates were used where indicated.

### Cell culture

Human PBMCs were isolated from the peripheral blood of patients and controls using Ficoll-Paque PLUS (Cytiva, Marlborough, MA, USA) followed by standard operating procedures. PBMCs were cultured in Roswell Park Memorial Institute (RPMI) 1640 media (Gibco, Gaithersburg, MD, USA) supplemented with 10% fetal bovine serum (FBS) (Gibco, Gaithersburg, MD, USA) and 1% penicillin and streptomycin (Gibco, Gaithersburg, MD, USA). Calvarial osteoblasts were prepared and cultured from postnatal day 1 mouse calvariae. The calvariae were digested in PBS (HyClone, Logan, UT, USA) containing 0.02% type II collagenase (Sigma Aldrich, St. Louis, MO, USA) and 0.05% trypsin for 10 min at 37 °C with shaking. BMSCs and BMMs were obtained from cultures of bone marrow collected from 4- to 6-week-old female mouse tibias and femurs as described^53^. Calvarial osteoblasts, BMSCs and BMMs were cultured in α-MEM (Gibco, Gaithersburg, MD, USA) supplemented with 10% FBS (Gibco, Gaithersburg, MD, USA) and 1% penicillin/streptomycin (1% p/s) (Gibco, Gaithersburg, MD, USA). All cells were cultured at 37 °C in a humidified 5% CO_2_ incubator. FBS was heat-inactivated before use.

### WES

A Blood Genomic DNA Mini Kit (Qiagen, Hilden, Germany) was used to extract genomic DNA from participant peripheral blood samples. The concentration and purity of the gDNA were determined by a spectrophotometer (NanoDrop, Thermo Fisher Scientific, Waltham, MA, USA). WES was further used to detect potential causal single nucleotide variants, insertions, and deletions (indels) in 4 patients (II4, III6, III10, IV5) and 4 controls (II5, II10, III5, III8) in the family. The hybridization capture procedure was performed with an Agilent SureSelect Illumina NovaSeq 6000 instrument (Illumina, San Diego, CA, USA) with the 180–200 bp paired-end read module. The mean coverage depth was 100×, with ~99.5% coverage of the target region. Clean reads were aligned to the human reference genome (hg19) using the Burrows–Wheeler Aligner (BWA; v.0.7.12). Whole-genome SNP scanning was performed using the Illumina Asian Screening Array. Linkage analysis was performed by the parametric linkage analysis package of MERLIN (ver. 1.1.2). Variants were filtered by the following criteria. Variants in the intronic, upstream, and downstream sites were removed, and those in the exonic and splicing functional regions were retained. New variants were classified according to the American College of Medical Genetics (ACMG) and Genomics criteria as being pathogenic, likely pathogenic, or of uncertain clinical significance, and variants classified as benign, or likely benign, were excluded. Variants were not considered if they had a read depth < 5× and a minor allele frequency (MAF) > 0.005 in databases including the dbSNP138, 1000 Genomes Project, Complete Genomics 69, Exome Variant Server, and Exome Sequencing Project databases. To assess the variant novelty, the Human Gene Mutation Database and ClinVar were used. GDD patients exhibit AD inheritance, so autosomal recessive (AR) inheritance pattern variants and variants carried by normal controls were also excluded. Thus, 9 new genetic variants were considered after the initial filtering step, which is listed in Supplementary Table 1.

### Sanger sequencing

Primer3 software was used to design primers for the 9 pathogenic variants detected. Nine variants were subsequently validated by Sanger sequencing of 2 controls (II2, II7). According to the GDD AD inheritance pattern, variants carried by normal controls were also excluded, and *ANO5*, *NANOS1*, and *WDR90* gene variants were determined. Again, we performed Sanger sequencing to confirm the 3 variants in 1 patient (IV6) and 2 other controls (III14, III15). After filtering the criteria, the new *ANO5* variant was ultimately considered a disease-causing mutation, and the results and detailed primers of Sanger sequencing are listed in Supplementary Tables 2 and 4.

### µCT

µCT of mandibles and femurs was performed using a micro-CT (Skyscan1176, USA, Bruker Siemens Inveon, Eschborn, Germany) with the following parameter settings: source voltage, 50 kV; source current, 450–500 μA; AI, 0.5 mm filter; pixel size, 9 μm; rotation step, 0.4°. Mandibles and femurs from 4- and 8-week-old male WT mice and *Ano5*^*−/−*^ mice were dissected, cleaned of soft tissue, and wrapped in PBS-soaked gauze. Threshold segmentation of bone from marrow and soft tissue was performed in conjunction with a constrained Gaussian filter to reduce noise. Mandibular bone parameters were measured by analyzing ~400 slices of trabecular bone under the first molar. Trabecular bone parameters were measured by analyzing 201 slices of the proximal metaphysis. Cortical bone parameters were measured by analyzing 51 slices in the mid-diaphysis, where the central portion was between the proximal and distal ends of the femur. Analyses were performed in agreement with guidelines for the assessment of bone microstructure in rodents using µCT^54^.

### Histology and histomorphometry

Histology and histomorphometry were performed as previously described^55^. Briefly, the mouse mandibles and femurs from 8-week-old mice were fixed with 10% neutral buffered formalin followed by decalcification in 10% EDTA for 3 weeks, paraffin embedding, TRAP staining and OCN immunohistochemistry. OCN was detected using a polyclonal OCN antibody (Cat# 23418-1-AP, 1:500 dilution, Proteintech, Sankt Leon-Rot, Germany) for 2 h, followed by Alexa 488-labeled secondary antibody (1:200 dilution, Cell Signaling Technology, Danvers, MA, USA) for 60 min. As indicated, DAPI staining (Sigma Aldrich, St. Louis, MO, USA) was also performed. Undecalcified femurs were embedded in paraffin for sectioning. Von Kossa staining was performed to determine osteoblast and osteoclast parameters. To determine the MAR, 25 mg/kg Alizarin red (Sigma Aldrich, St. Louis, MO, USA) was injected at 14 days, and 20 mg/kg calcein (Sigma Aldrich, St. Louis, MO, USA) was injected 7 days before bone collection. The indices were measured and analyzed using Bioquant Osteo 2009 v9.0 (Bioquant, Nashville, TN, USA). Bone histomorphometric parameters were calculated according to the standardized nomenclature for bone histomorphometry^56^. Pannoramic 250 Flash was used for slide scanning (3DHISTECH, Budapest, Hungary). An Olympus Fluoview 1000 laser confocal imaging system (Olympus, Shinjuku City, Tokyo, Japan) was used for microscope photography. Histological viewing and analysis were performed with Case Viewer (3DHISTECH, Budapest, Hungary) and ImageJ2/FIJI (NIH).

### Cell proliferation assay

Cells were seeded at 1 × 10^4^ cells per well in 96-well plates, and proliferation was determined using the cell proliferation reagent Cell Counting Kit-8 (Dojindo, Kumamoto, Japan) according to the manufacturer’s instructions.

### In vitro osteoclast differentiation

Mature osteoclasts were generated as previously described^57^. Mouse BMMs or human PBMCs were cultured in α-MEM (Gibco, Gaithersburg, MD, USA) containing 10% FBS (Gibco, Gaithersburg, MD, USA) supplemented with 30 ng/mL recombinant mouse/human M-CSF and 50 ng/mL recombinant mouse/human RANKL (R&D Systems, Minneapolis, MN, USA) for 5–7 days in tissue culture dishes to induce osteoclast formation. Mature osteoclasts were characterized by staining for TRAP activity using the Acid Phosphatase Leukocyte Kit (Sigma Aldrich, St. Louis, MO, USA), and TRAP-positive multinucleated cells (>3 nuclei/cell) were counted.

### In vitro osteoblast differentiation

For osteogenesis, BMSCs or calvarial osteoblasts were seeded at 2.5 × 10^4^ cells per cm^2^ in 6- or 48-well tissue culture plates. The next day, the cells were transferred to osteogenic media: α-MEM medium (Gibco, Gaithersburg, MD, USA) supplemented with 10% FBS (Gibco, Gaithersburg, MD, USA), 1% P/S (Gibco, Gaithersburg, MD, USA), 50 µg/mL l-ascorbic acid (Sigma Aldrich, St. Louis, MO, USA) and 5 mM β-glycerol phosphate (Sigma Aldrich, St. Louis, MO, USA). RNA isolation was performed at 0, 3 and 7 days. ALP staining was performed at 7 days using the BCIP/NBT Alkaline Phosphatase Color Development Kit (Beyotime, Shanghai, China), and Alizarin red staining (Sigma Aldrich, St. Louis, MO, USA) of minerals was conducted at 21 days according to the manufacturer’s instructions.

### Lentiviral gene transduction

The *Ano5* gene was subcloned into the pCDH-CMV-MCS-EF1-GFP-puro (CD513B-1) lentiviral vector by Shanghai IBS Biological Technology Co., Ltd. according to the manufacturer’s instructions. *Ano5*^*−/−*^ BMMs and calvarial osteoblasts were transduced with recombinant lentiviral particles in the presence of 8 mg/mL polybrene (IBS Bio, Shanghai, China). Two days after lentivirus transduction, the cells were selected for approximately one week with 2–4 µg/mL puromycin (Sigma Aldrich, St. Louis, MO, USA) in the culture media. The expression of the recombinant RNA was confirmed by qPCR.

### RNA extraction and gene expression analysis

RNA was isolated from cultured cells with TRIzol reagent (Invitrogen, Carlsbad, CA, USA) and purified using an AxyPrep Multisource RNA Miniprep Kit (Axygen, USA). RNA quality was confirmed using a Nanodrop spectrophotometer (Thermo Fisher Scientific, Waltham, MA, USA). Mouse complementary DNA (cDNA) was reverse-transcribed from 1 μg total RNA with PrimeScript RT Master Mix (Takara Bio, Kusatsu, Shiga, Japan). qPCR was performed in triplicate using TB Green Premix Ex Taq (Takara Bio, Kusatsu, Shiga, Japan) according to the manufacturer’s instructions. For each transcript examined, mRNA expression was normalized to *Actin*. Primer sequences for each gene are provided in Supplementary Table 3.

### Calcium oscillations

A total of 1 × 10^4^ BMMs or human PBMCs were seeded on 35-mm confocal dishes and cultured with 30 ng/mL M-CSF (R&D Systems, Minneapolis, MN, USA) in the presence of 50 ng/mL RANKL (R&D Systems, Minneapolis, MN, USA) or without RANKL as controls for 72 h. BMSCs (2 × 10^4^) were cultured on confocal dishes with osteogenic media for 7 days. The assay buffer consisted of Hank’s buffered salt solution (Gibco, Gaithersburg, MD, USA, without CaCl_2_), 2% FCS, and 1 mM probenecid solution (Invitrogen, Carlsbad, CA, USA) (pH 7.4). The cells were then incubated in the presence of 5 µM Fluo-4 AM (Invitrogen, Carlsbad, CA, USA) and 0.05% Pluronic F-127 (Invitrogen, Carlsbad, CA, USA) in the dark at 37 °C for 45 min in assay buffer. The cells were washed twice with assay buffer and then incubated in fresh assay buffer for 20 min. The cells were viewed on the inverted stage of a Leica TCS SP8 confocal microscope (Leica Biosystems, Wetzlar, Germany). At an excitation wavelength of 488 nm, Fluo-4 was analyzed simultaneously at 5 s intervals for BMMs or human PBMCs and at 10 s intervals for BMSCs. The increase from the basal level was determined by adding 10 μM ionomycin (Abcam, Cambridge, UK). Peak analysis was performed to obtain parameters with ImageJ2/FIJI (NIH).

### Flow cytometric detection of phosphatidylserine (PS)

To induce PS translocation, BMMs and primary osteoblasts were seeded at a density of 10 × 4 cells/cm^2^, cultured, and differentiated for 48 h and 7 days, respectively. Cells were harvested by trypsinization, washed first in growth medium and then washed in PBS (HyClone, Logan, UT, USA) supplemented with 0.5 mM CaCl_2_. Subsequently, the cells were treated with 10 mM ionomycin (Abcam, Cambridge, UK) or ethanol for 5 min or 2.5 μM staurosporine (Abcam, Cambridge, UK) for 8 h at 37 °C. Ionomycin was removed by washing in ice-cold PBS. Treated cells were then washed in annexin-binding buffer and stained with Alexa Fluor^®^ 488 annexin V and propidium iodide (PI) (Invitrogen, Carlsbad, CA, USA) according to standard operating procedures. Gating strategy: (1) implement FSC/SSC gate for living cells, (2) define the gate for FITC- or FITC + cells, (3) apply this gate to all samples. Flow cytometric analysis was conducted using a BD LSR Fortessa (Becton Dickinson, Heidelberg, Germany), and the data were processed with FlowJo v.10.7.

### Confocal fluorescence microscopy

Cells were seeded in cell culture dishes with glass bottoms (Greiner Bio, Monroe, NC, USA). Live cell imaging was performed using a Leica TCS SP8 confocal laser scanning microscope (Leica Biosystems, Wetzlar, Germany) equipped with ×40 oil immersion objectives. Final images represent the average of 4–12 acquisitions. No filtering was applied.

### Western blot analysis

Cells were lysed in RIPA buffer in the presence of phosphatase and protease inhibitor cocktails (Thermo Fisher Scientific, Waltham, MA, USA). A standard protocol was used for Western blotting. In brief, samples were separated on 4–20% SurePage gels (GenScript, Piscataway, NJ, USA) and transferred onto a 0.45-μm PVDF membrane (Thermo Fisher Scientific, Waltham, MA, USA). Antigen detection was performed using antibodies directed against GAPDH (1:1000 dilution, Cat# 5174), β-Actin (1:1000 dilution, Cat# 3700), β-Catenin (1:1000 dilution, Cat# 8480), NFATc1 (1:1000 dilution, Cat# 8032), c-Fos (1:1000 dilution, Cat# 2250), GSK-3β (1:1000 dilution, Cat# 12456), Axin1 (1:1000 dilution, Cat# 2087), and Dvl2 (1:1000 dilution, Cat# 3224) from Cell Signaling Technology, Danvers, MA, USA; WNT1 (1:1000 dilution, Cat# abs131412) from Absin Bioscience Inc., Shanghai, China; ANO5/TMEM16E (1:100 dilution, Cat# LS‑C330335) from LSBio, Seattle, WA, USA. The bound primary antibodies were detected with anti-mouse IgG (H + L) (Cat# 5257) and anti-rabbit IgG (H + L) (DyLight™ 800 4× PEG Conjugate) (Cat# 5151) (diluted to 1:2000; Cell Signaling Technology, Danvers, MA, USA) using the Odyssey CLx system (LI-COR Biosciences, Lincoln, NE, USA). Western blot images were quantified with Image Studio Lite (LI-COR Biosciences, Lincoln, NE, USA). All blots or gels were derived from the same experiment and processed in parallel. Uncropped and unprocessed scans of blots in this study are provided in Supplementary Fig. 5.

### Statistical analysis

Statistical analysis was performed using GraphPad Prism 9.0 software. We used the unpaired two-tailed Student’s *t* test for comparisons between two groups and the multiple *t* test or two-way ANOVA with Tukey’s correction for multiple comparisons. Bar graphs present the mean ± standard error of the mean (SEM). No statistical methods were used to determine the sample size, and incomplete data were excluded from the analysis performed in this manuscript. Statistical details about the sample size (*n*) and *p* value are reported in the figure legends.

### Reporting summary

Further information on research design is available in the Nature Research Reporting Summary linked to this article.

## Supplementary information

Supplements

Reporting Summary Checklist

## Data Availability

In accordance with informed consent signed by participants, sharing of supporting details is subject to ethical review by the Institutional Ethics Committee of Ninth People’s Hospital. The data that support the findings of this study are available from the corresponding author (X.J.Q.) upon reasonable request. The whole-exome sequencing and Sanger-sequencing data of all patients and controls have been deposited in the Sequence Read Archive (SRA) under the primary accession code PRJNA817717.
