# Supplementary material for: Ano5 modulates calcium signaling during bone homeostasis in gnathodiaphyseal dysplasia
Source: NPJ Genom Med. 2022 Aug 18;7:48. doi: 10.1038/s41525-022-00312-1 (PMC9388649; doi:10.1038/s41525-022-00312-1)
Supplement: Supplementary file 1 — Supplements [file 41525_2022_312_MOESM1_ESM.pdf]

Supplementary Figures and Figure Legends

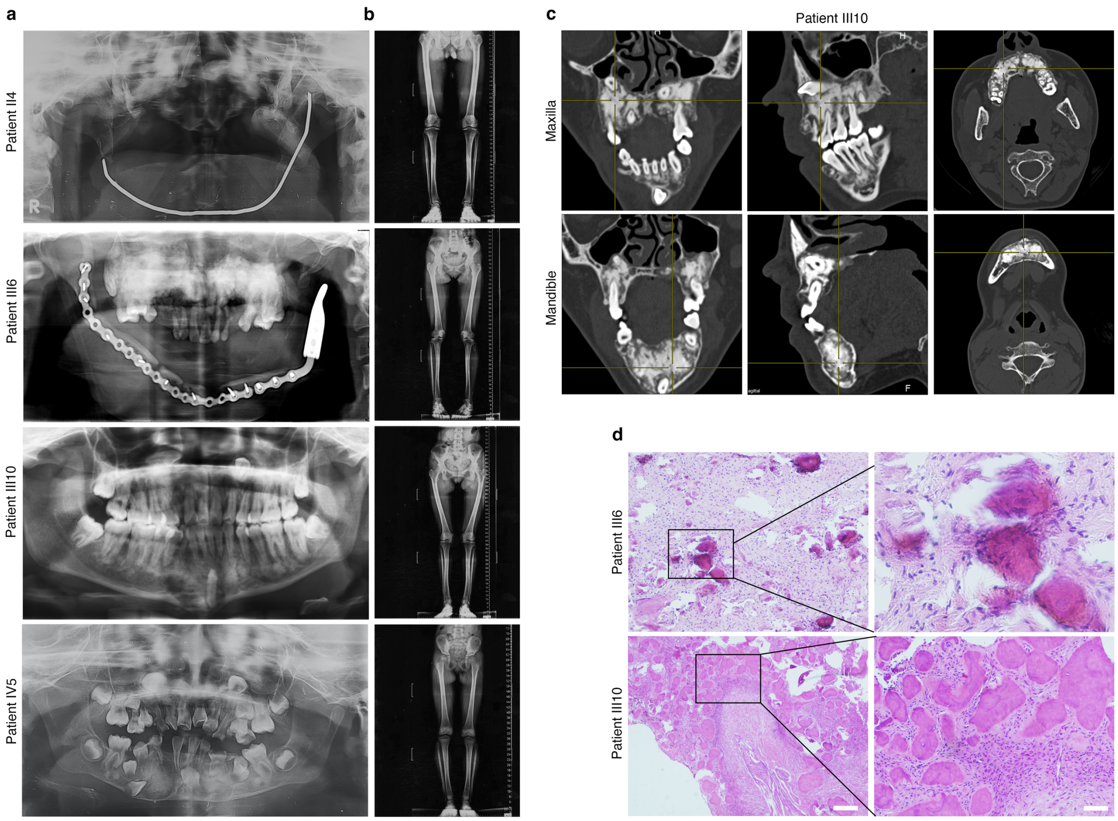

Supplementary Fig. 1 Clinical follow-up of GDD patients in the family. a orthopantomography and **b** radiographs of the upper and lower limbs of the patients (II4, III6, III10, IV5). Facial deformity characteristics, missing and displaced teeth, diaphyseal cortical thickening and mild bowing of tubular bones in patients were shown. **c** Histologic findings of the maxilla lesions in the proband III6, and the tooth apex in patient III10. Scale bar, 100 μm (main), 50 μm (insets). **d** CT image of patient III10 showing jaw lesions occupying alveolar processes involving the bilateral mandibular body and the entire maxilla.

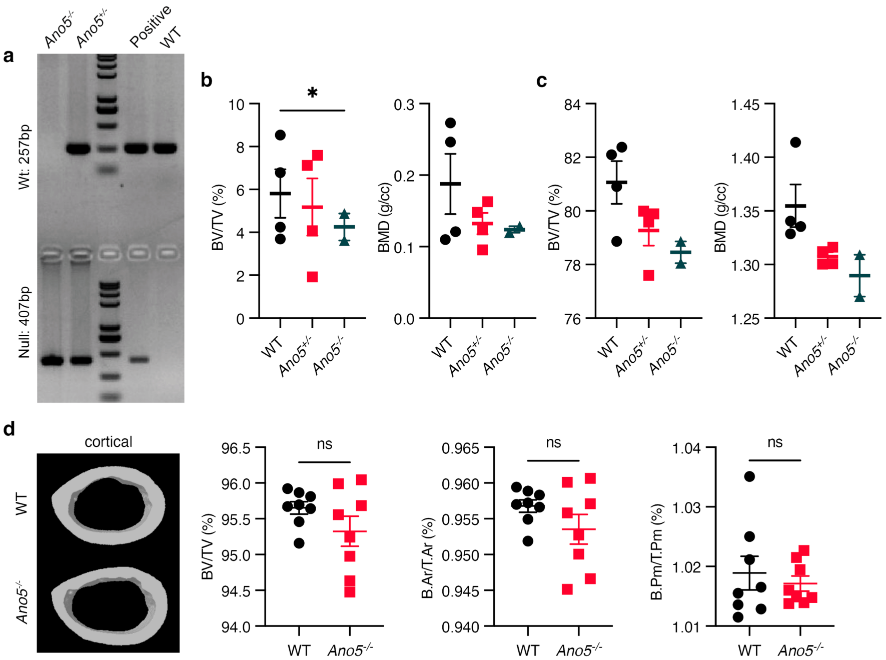

Supplementary Fig. 2 Ablation of *Ano5* decreased bone density in mice. a RT-PCR genotyping analysis of WT, *Ano5*$^{+/-}$, and *Ano5*$^{-/-}$ mice. **b** Proximal femur trabeculae and **c** mandible trabeculae quantification of BV/TV and BMD as determined by μCT of 4-week-old WT, *Ano5*$^{+/-}$ and *Ano5*$^{-/-}$ male mice (n=4). **d** 3D reconstruction of the cortex at the femoral midshaft of 8-week-old WT and *Ano5*$^{-/-}$ male mice, and BV/TV, B.Ar/T.Ar, and B.Pm/T.Pm were quantified (n=8). Data are represented as mean ± SEM. Two-tailed Student's t test (for **d**) and two-way ANOVA with Tukey's correction comparisons test (for **b, c**). *$P < 0.05$; ns: not significant $P>0.05$.

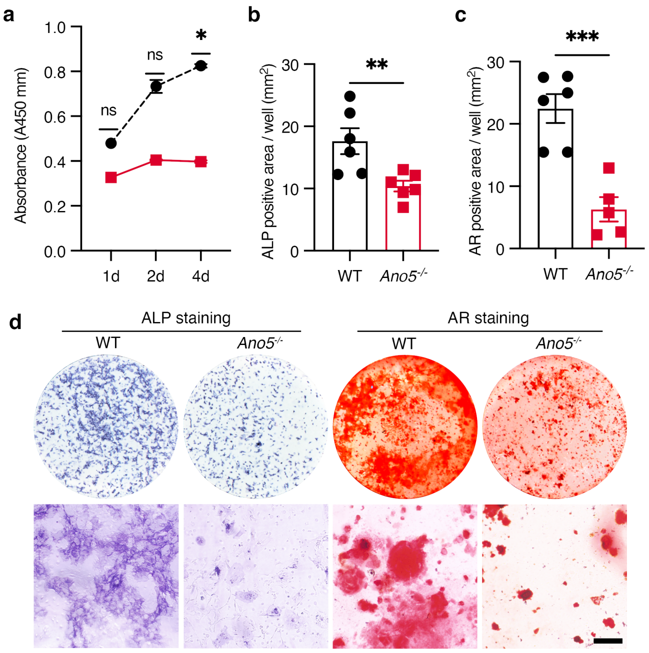

Supplementary Fig. 3 Ablation of *Ano5* inhibited BMSC-derived osteoblast differentiation. a The proliferation of WT and *Ano5*$^{-/-}$ BMSCs according to CCK-8 assay. **b, c** quantification and **d** representative images of ALP staining and AR staining after 7 and 21 days of osteogenic differentiation in WT and *Ano5*$^{-/-}$ BMSCs (n=6). Scale bar, 50 μm. All results are representative of data generated from at least three independent experiments. Data are represented as mean ± SEM. Two-tailed Student's t test comparisons test. *P < 0.05, **P < 0.01, ***P < 0.001; ns: not significant P>0.05.

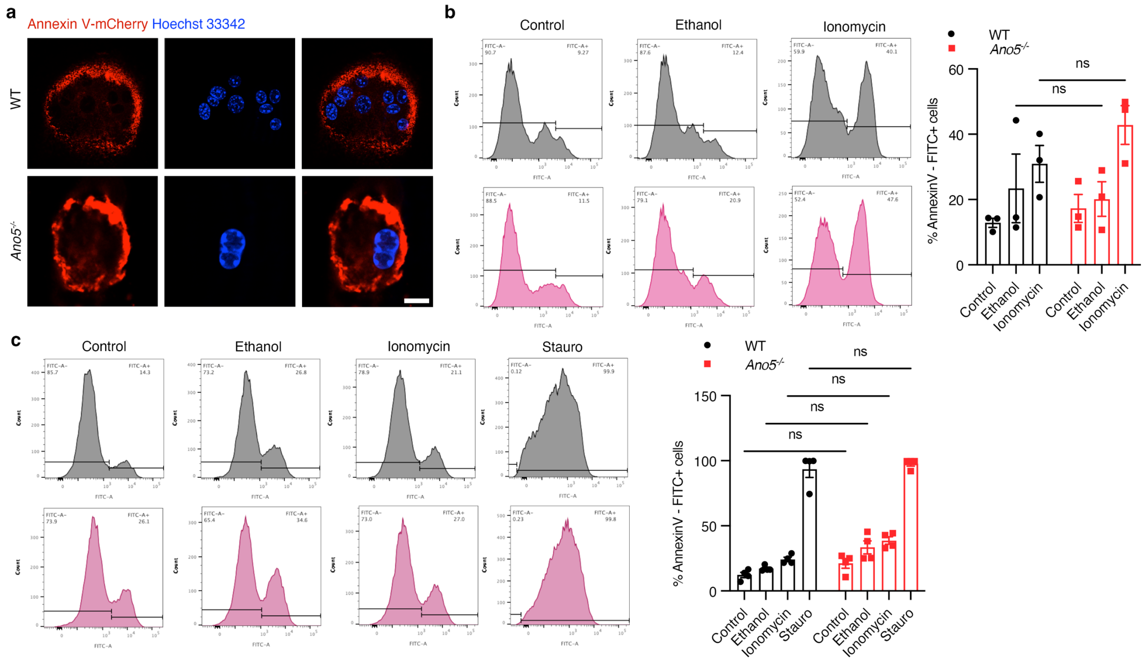

Supplementary Fig. 4 Phospholipid scrambling of TMEM16E. a Confocal images of live WT and *Ano5*$^{-/-}$ osteoclasts treated with 10 mM ionomycin for 5 min and stained with Annexin V-mCherry. Scale bar, 20 µm. **b** WT and *Ano5*$^{-/-}$ osteoclasts were treated with Ethanol or 10 mM ionomycin for 5 minutes and stained with fluorescence-labeled annexin V (Alexa Fluor® 488, FITC) to detect cell surface exposure of PS followed by FACS analysis and quantification. **c** WT and *Ano5*$^{-/-}$ osteocblasts were treated with Ethanol or 10 mM ionomycin for 5 minutes or 2.5µM staurosporine for 8h and stained with fluorescence-labeled annexin V followed by FACS analysis and quantification. All results are representative of data generated from at least three independent experiments. Data are represented as mean ± SEM. Two-way ANOVA with Tukey’s correction comparisons test. ns: not significant P>0.05.

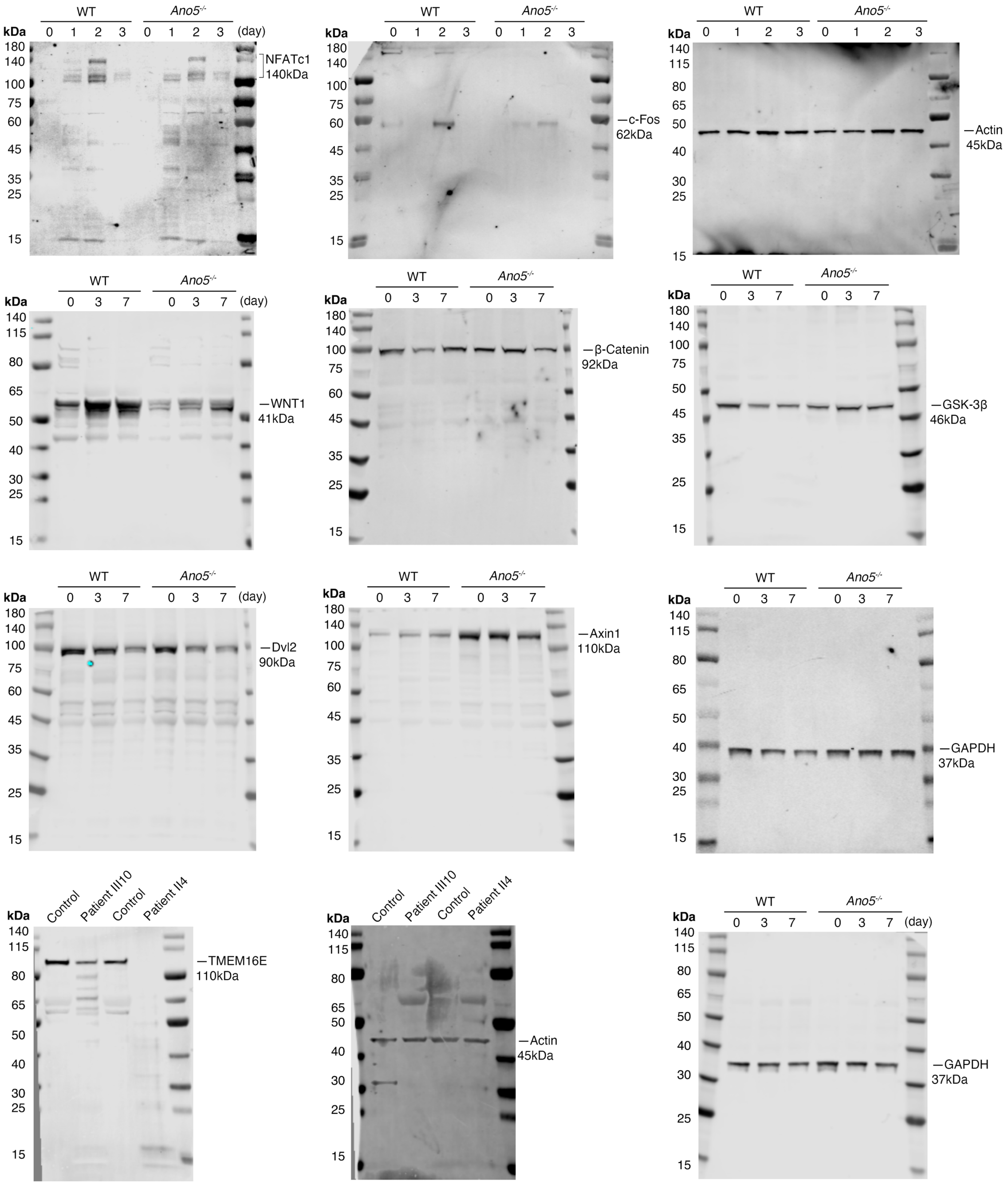

Supplementary Fig. 5 Uncropped Western blotting images used in this study.

Supplementary Tables

Supplementary Table 1. Full list of genetic variants after initial filtering of the whole-exome sequencing (WES) results.

Gene	Chro position	Het	AAChange	1000genome	ExAC	InterVar	SIFT	Polyphen2	PROVEAN	CADD
AARS2	chr6-44278169	het	c.761G>C, p.G254A	0.0004	0.0001	Uncertain significance	D	D	D	24.9
ANO5	chr11-22272353	het	c.1077_1078insGATTATTGGAGACTAAATAGTACGTGTTTG, p.L369_A370insDYWRLNSTCL	-	-	-	-	-	-	-
CLSTN1	chr1-9794099	het	c.2155G>A, p.V719M	-	-	Uncertain significance	D	D	N	24.2
FAT1	chr4-187539275	het	c.8465T>C, p.L2822P	0.0016	0.0007	Uncertain significance	D	P	D	25.8
LRP2BP	chr4-186295326	het	c.542T>C, p.L181P	-	-	Uncertain significance	D	D	D	24.7
NANOS1	chr10-120789812	het	c.499_500insCCG, p.A173_T174insA	0.0004	-	-	-	-	-	-
PRDM15	chr21-43298845	het	c.371dupG, p.P125Sfs*8	-	0.0005	-	-	-	-	-
USP54	chr10-75258556	het	c.4886C>G, p.P1629R	-	0.0000	Uncertain significance	D	P	D	22.6
WDR90	chr16-703802	het	c.1436C>T, p.T479M	-	0.0000	Uncertain significance	D	D	D	24.4

Chro=chromosome number; Ref=reference allele; Het=inheritance pattern; AA=amino acid; P=possibly damaging; B=benign; D=deleterious/damaging

Supplementary Table 2. Full list of genetic variants from the Sanger sequencing results.

Gene	Chro position	AAChange	II 2	II 7	III 14	III 15	IV 6
AARS2	chr6-44278169	c.761G>C, p.G254A	het	het	-	-	-
ANO5	chr11-22272353	c.1077_1078insGATTATTGGAGACTAAATAGTACGTGTTTG, p.L369_A370insDYWRLNSTCL	N	N	N	N	het
CLSTN1	chr1-9794099	c.2155G>A, p.V719M	het	N	-	-	-
FAT1	chr4-187539275	c.8465T>C, p.L2822P	het	het	-	-	-
LRP2BP	chr4-186295326	c.542T>C, p.L181P	het	het	-	-	-
NANOS1	chr10-120789812	c.499_500insCCG, p.A173_T174insA	N	N	-	-	N
PRDM15	chr21-43298845	c.371dupG, p.P125Sfs*8	het	het	-	-	-
USP54	chr10-75258556	c.4886C>G, p.P1629R	N	het	-	-	-
WDR90	chr16-703802	c.1436C>T, p.T479M	N	N	het	het	het

N=not found; het=heterozygote

Supplementary Table 3. Full list of mouse primers for qPCR.

Primer name	Primers (5’-3’)
Ocn forward	GCAATAAGGTAGTGAACAGACTCC
Ocn reverse	CCATAGATGCGTTTGTAGGCGG
Spp1 forward	GCTTGGCTTATGGACTGAGGTC
Spp1 reverse	CCTTAGACTCACCGCTCTTCATG
Trap forward	GCGACCATTGTTAGCCACATACG
Trap reverse	CGTTGATGTCGCACAGAGGGAT
Nfatc1 forward	GGTGCCTTTTGCGAGCAGTATC
Nfatc1 reverse	CGTATGGACCAGAATGTGACGG
Ctsk forward	AGCAGAACGGAGGCATTGACTC
Ctsk reverse	CCCTCTGCATTTAGCTGCCTTTG
Cfos forward	GGGAATGGTGAAGACCGTGTCA
Cfos reverse	GCAGCCATCTTATTCCGTTCCC
Ano5 forward	ACACATGCCTCTATGCCATCGC
Ano5 reverse	GGCAAAGACTGACAGGCGGTAT
Gapdh forward	CATCACTGCCACCCAGAAGACTG
Gapdh reverse	ATGCCAGTGAGCTTCCCGTTCAG
Actin forward	CATTGCTGACAGGATGCAGAAGG
Actin reverse	TGCTGGAAGGTGGACAGTGAGG

Supplementary Table 4. Full list of human primers for Sanger Sequencing.

Primer name	Primers (5’-3’)
AARS2-p78169-F	GATACTGATTGTCTGGTTCCTCCC
AARS2-p78169-R	GTATGGCGTTGAGCAGCG
ANO5-p72353-F	GCTGGGCTCTGAAAACTCTACT
ANO5-p72353-R	TTTCTTTATGGGACTATTTACTCACC
CLSTN1-p94099-F	GCCCCACTGCCCCTACTT
CLSTN1-p94099-R	CCTTCCCCATCTCCCTCTAA
FAT1-p39275-F	CAGACAGAGCGGGAGACTGAAGTT
FAT1-p39275-R	TGACATCGGTGACGGTAACATC
LRP2BP-p95326-F	CTGAGAACAAGATTAGGTTTGAAGG
LRP2BP-p95326-R	TAGAGGTAGGGAAGGGGACATT
L1039-NANOS1-812-F3	GCTGGAATTGCGCGCGCTGGAGCT
L1039-NANOS1-812-R3	GCACGGGACACAGCACTCGCCCGTC
PRDM15-p98845-F	GAGGCCCCAGGGGATGTCGTTA
PRDM15-p98845-R	ACCTTCTCTCTGCCACCAAAA
USP54-p58556-F	CCCACCCTCCCATTGTTCA
USP54-p58556-R	ACCCCTCTCCCACTGTTCTC
WDR90-p03802-F	GGTGCTTGTGCCTGTTCCG
WDR90-p03802-R	TGGCTTAAACCGACTCCGATG
